# 600. Rapid Detection of Antibiotic Resistance Genes Direct from Whole Blood Samples by T2 Magnetic Resonance (T2MR^®^)

**DOI:** 10.1093/ofid/ofad500.667

**Published:** 2023-11-27

**Authors:** Jin Wang, Christopher Cacciatore, Jessica L Snyder, Heather Noucas, Roger Smith

**Affiliations:** T2 Biosystems, Inc., Lexington, Massachusetts; T2 Biosystems, Inc., Lexington, Massachusetts; T2 Biosystems, Inc, Lexington, Massachusetts; T2 Biosystems, Inc., Lexington, Massachusetts; T2 Biosystems, Inc, Lexington, Massachusetts

## Abstract

**Background:**

According to the Centers for Disease Control and Prevention (CDC), more than 2.8 million antibiotic-resistant infections occur in the US each year and more than 35,000 individuals die due to these infections. Rapid identification of drug resistance marker(s) in blood specimens may aid clinicians in getting patients on appropriate antimicrobial treatment early and decrease associated morbidity and mortality.

**Methods:**

T2Resistance Panel is a qualitative, multiplex, T2MR assay designed to identify molecular markers of antimicrobial resistance in human whole blood specimens. The Panel detects thirteen molecular markers of resistance: *bla*_KPC_, *bla*_CTX-M 14_, *bla*_CTX-M 15_, *bla*_NDM_, *bla*_VIM_, *bla*_IMP_, *bla*_OXA-48 Group_, *vanA, vanB, mecA, mecC*, *bla*_CMY_ and *bla*_DHA_. Bacterial isolates spiked into whole blood were used to evaluate analytical sensitivity, specificity and reactivity. When possible, testing utilized different bacterial species or gene variants. Assay performance was also assessed in the presence of potential interfering substances.

**Results:**

The average time to result was approximately 3.5 hours. A Limit of Detection of < 20 CFU/mL was demonstrated for all resistance markers. The assay successfully detected drug resistance markers from genetically and geographically diverse strains. There was no cross reactivity observed, and the presence of common interferents did not affect the detection of drug resistance markers.

**Conclusion:**

The T2Resistance Panel is a rapid and sensitive assay that detects 13 antimicrobial resistance markers directly from whole blood specimens. Rapid detection of these resistance markers may allow clinicians to provide targeted and effective antimicrobial therapy earlier to patients.

This project has been supported in whole or in part with federal funds from the Department of Health and Human Services; Administration for Strategic Preparedness and Response; Biomedical Advanced Research and Development Authority (BARDA), under contract number 75A50119C00053. The findings and conclusions in this presentation have not been formally disseminated by the Department of Health and Human Services and should not be construed to represent any agency determination or policy.

**Disclosures:**

**Jin Wang, PhD**, T2 Biosystems, Inc.: Stocks/Bonds **Christopher Cacciatore, BS**, T2 Biosystems, Inc.: Stocks/Bonds **Jessica L. Snyder, Ph.D.**, T2 Biosystems, Inc.: Stocks/Bonds **Heather Noucas, MS**, T2 Biosystems, Inc.: Stocks/Bonds **Roger Smith, Ph.D.**, T2 Biosystems, Inc.: Stocks/Bonds

